# Mechanical Dispersion of Nanoparticles and Its Effect on the Specific Heat Capacity of Impure Binary Nitrate Salt Mixtures

**DOI:** 10.3390/nano5031136

**Published:** 2015-06-29

**Authors:** Mathieu Lasfargues, Qiao Geng, Hui Cao, Yulong Ding

**Affiliations:** 1 School of Chemical and Process Engineering, University of Leeds, Leeds LS2 9JT, UK; E-Mail: m.lasfargues@leeds.ac.uk; 2 School of Chemical Engineering, University of Birmingham, Birmingham B15 2TT, UK; E-Mails: gxq471@student.bham.ac.uk (Q.G.); h.cao@bham.ac.uk (H.G.)

**Keywords:** nanoparticles, molten salt, energy storage

## Abstract

In this study, the effect of nanoparticle concentration was tested for both CuO and TiO_2_ in eutectic mixture of sodium and potassium nitrate. Results showed an enhancement in specific heat capacity (*C_p_*) for both types of nanoparticles (+10.48% at 440 °C for 0.1 wt % CuO and +4.95% at 440 °C for 0.5 wt % TiO_2_) but the behavior toward a rise in concentration was different with CuO displaying its highest enhancement at the lowest concentration whilst TiO_2_ showed no concentration dependence for three of the four different concentrations tested. The production of cluster of nanoparticles was visible in CuO but not in TiO_2_. This formation of nanostructure in molten salt might promote the enhancement in *C_p_*. However, the size and shape of these structures will most likely impact the energy density of the molten salt.

## 1. Introduction

The addition of nanoparticles into a base liquid forms a two-phase mixture termed nanofluid by Choi in 1995 [1]. With nominal size ranging from 1 to 100 nm, these nanoparticles have been reported to display anomalous enhancement of the thermal characteristics of the fluid within which they are suspended. The addition of varied fraction of nanoparticles (Al_2_O_3_, Au, Cu, TiO_2_, CuO, carbon nanotube…) into base fluid such as pumped oil, glycerol, toluene, water have shown enhanced thermal conductivity with minutes addition (maximum of 5.0 vol. %) [2,3,4,5,6,7,8,9,10,11]. The multifaceted nature of nanofluids has been difficult to model and reported data have shown that various factors such as size of particle, concentration, particle surface properties, shape, interaction between base liquid and particle could affect the thermal characteristics in different manners [3,8,12,13,14,15,16,17,18,19,20]. With the literatures been full of papers reporting these abnormal enhancements in thermal conductivity, recent theory and understanding points toward the effective medium theory by Maxwell as a good model for the prediction of thermal conductivity [21,22]. However, very little experiments in comparison have been conducted on heat capacity and modelling of non-ionic fluids using classical and statistical model has shown good agreement with the obtained data following the equation below [23]:

$$C_{p,nf}=\;\frac{\emptyset {\left(\rho C_{p}\right)}_{n}+\;\left(1\;-\;\emptyset \right){\left(\rho C_{p}\right)}_{f}}{\emptyset \rho_{n}+\;(1\;-\;\emptyset )\rho_{f}}$$

where *C_p_*, Ø, ρ, *nf*, *n* and *f* respectively represent specific heat capacity, volume fraction, density, nanofluid, nanoparticle and fluid. It has implied that increased thermal conductivity has been translated into decrease in specific heat capacity as shown by Pantzali *et al*. [24]. However this has not been the case in ionic fluids such as molten salt where the addition of nanoparticles into this liquid base has promoted an enhancement in specific heat capacity which does not follow the above equation. Indeed various publications have demonstrated a general rise in specific heat capacity when nanoparticles were added to the system [25,26,27,28,29,30,31]. This paper looks at the effect of various concentrations (0.1 wt % to 1.5 wt %) of copper oxide and titanium dioxide on the specific heat capacity, as well as the enthalpy of melting.

## 2. Experimental Section

### 2.1. Salt Production and Nanoparticles Dispersion

Sodium nitrate (FISHER, Loughborough, UK) (98% Pure) and potassium nitrate (SIGMA-ALDRICH, Suffolk, UK) (98% Pure) were purchased for these tests. Using a Ball-Mill Model (Pascall Engineering, Sussex, UK; BERCO, S/N 17520) with 9 mm stainless steel bearing, the eutectic mixture was grinded and mixed with the following ratio of salts (60 wt % NaNO_3_ + 40 wt % KNO_3_), melted at 300 °C for a few hours before been grinded again into fine powder. The purity of the sample was intentionally low to ensure it met similar criteria to that used in a concentrated solar power plant where it would not be economically viable to utilise high purity salt mixture.

Oxide nanoparticles of copper (QinetiQ Nanomaterials Limited, Farnborough, UK) and titanium (Nanophase NanoTek, Romeoville, IL, USA) with APS size of 29 nm and 34 nm respectively were dispersed into the binary mixture of nitrate using ball-mill with 9 mm stainless steel bearing.

Unlike other papers, the methodology does not involve the use of water to dissolve the salt and disperse the nanoparticle through sonication before re-crystallisation, drying and eventually melting is to take place. Even when using such methods, it has been showed that agglomeration of nanoparticles occurs. Here the dispersion is purely physical through the use of a ball-mill.

### 2.2. Differential Scanning Calorimeter (DSC)

Specific heat capacity (*C_p_*) and enthalpy of change were measured using heat-flux differential scanning calorimeter (DSC) with automated sampler (DSC 1, Mettler Toledo, Leicester, UK). The *C_p_* measurements were carried out through a standard procedure using a sapphire reference. The thermal cycle started with a 5 min isothermal at 250 °C followed by a dynamic phase with a ramp rate of 40 °C/min to 450 °C and another 5 min isothermal at 450 °C. Three samples were produced for each blend and each sample was consecutively tested three times.

Specific heat capacity was first calculated for sapphire in order to check the validity of the methodology. With variation obtained of the order of ±0.3% compared to that of the reference values, the approach was deemed accurate enough to start the tests on both CuO and TiO_2_ doped salt.

The samples were then loaded into 30 µL platinum crucibles (Mettler Toledo). The latter were placed onto a hot-plate and heated up to 250 °C for 30 min to allow the salt to melt before been loaded onto the carousel ready for testing.

### 2.3. Scanning Electron Microscopy (SEM) and Energy Dispersive X-Ray (EDX)

Morphological analysis of the samples was carried out using a field emission scanning electron microscopy Hitachi SU8230 (Hitachi, Berkshire, UK). Samples were mounted after DSC measurements on 12 mm diameter aluminium scanning electron microscopy (SEM) stub and coated with 3 nm of platinum/palladium mixture (80/20 Ratio) using a high resolution sputter coater.

## 3. Results and Discussions

**Figure 1:**
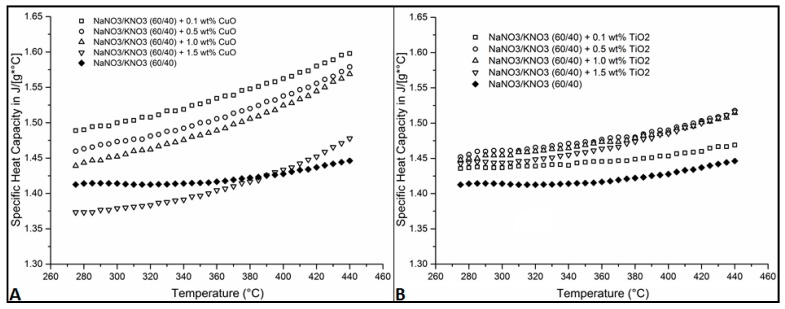
Specific heat capacity (*C_p_*) measurements: (**A**) 0.1 to 1.5 wt % CuO addition to 60/40 mixtures of Na/K-NO_3_; (**B**) 0.1 to 1.5 wt % TiO_2_ addition to 60/40 mixtures of Na/K-NO_3_.

The use of copper oxide revealed a concentration dependency where the lowest ratio (0.1 wt %) promoted the highest rise in *C_p_* (10.48% at 440 °C—Table 1) with a decrease seen as the amount added rose to 1.5 wt % (Figure 1A). This was also indicated in Table 1 where the percentage enhancement in *C_p_* decreases with a rise the concentration of CuO (10.48% for 0.1 wt %, 9.18% for 0.5 wt %, 8.49% for 1.0 wt % and 2.19% for 1.5 wt % CuO at 440 °C, see Table 1). The latter showed a detrimental behavior to the specific heat capacity (Figure 1A, Table 1—CuO). This has been reported by Chieruzzi *et al*. which showed negative enhancement when using 0.5 and 1.5 wt % SiO_2_ whilst 1.0 wt % SiO_2_ provided increase in *C_p_* indicating the need for an optimum ratio [32,33]. Unlike Chieruzzi’s research, test on TiO_2_ (Figure 1B) led to a similar rise in *C_p_* with 0.5, 1.0 and 1.5 wt % of TiO_2_ achieving a 5.0% increase at 440 °C (Table 1—TiO_2_) [32]. Presumably the different methodology in the dispersion of nanoparticles as well as size and production process of the nanoparticle itself would account for these differing results obtained.

**Table 1:** Percentage increase in specific heat capacity (*C_p_*) compared to base salt for different concentrations of CuO and TiO_2_.

Temperature	0.1 wt % CuO	0.5 wt % CuO	1.0 wt % CuO	1.5 wt % CuO	0.1 wt % TiO2	0.5 wt % TiO2	1.0 wt % TiO2	1.5 wt % TiO2
°C	% Increase	% Increase	% Increase	% Increase	% Increase	% Increase	% Increase	% Increase
265	4.87	2.97	1.47	−2.79	1.39	2.66	2.03	2.07
300	6.08	4.19	2.70	−2.49	1.60	3.33	2.86	2.10
350	7.90	6.02	4.76	−1.29	2.09	4.13	3.82	3.10
400	9.43	7.71	6.78	0.40	1.80	4.37	4.13	4.02
440	10.48	9.18	8.49	2.19	1.57	4.95	4.72	4.85

**Figure 2:**
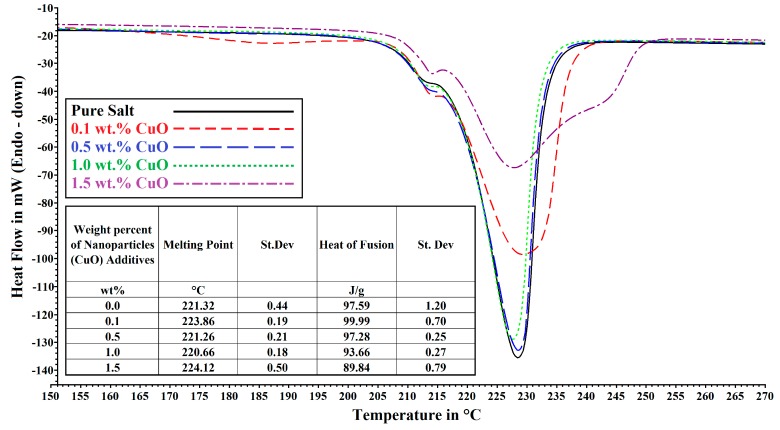
Differential scanning calorimetry (DSC) curves of NaNO_3_ and KNO_3_ eutectic mixture (60/40) with and without CuO nanoparticles. Table inserted in the graph shows the average melting point and heat of fusion values.

**Figure 3:**
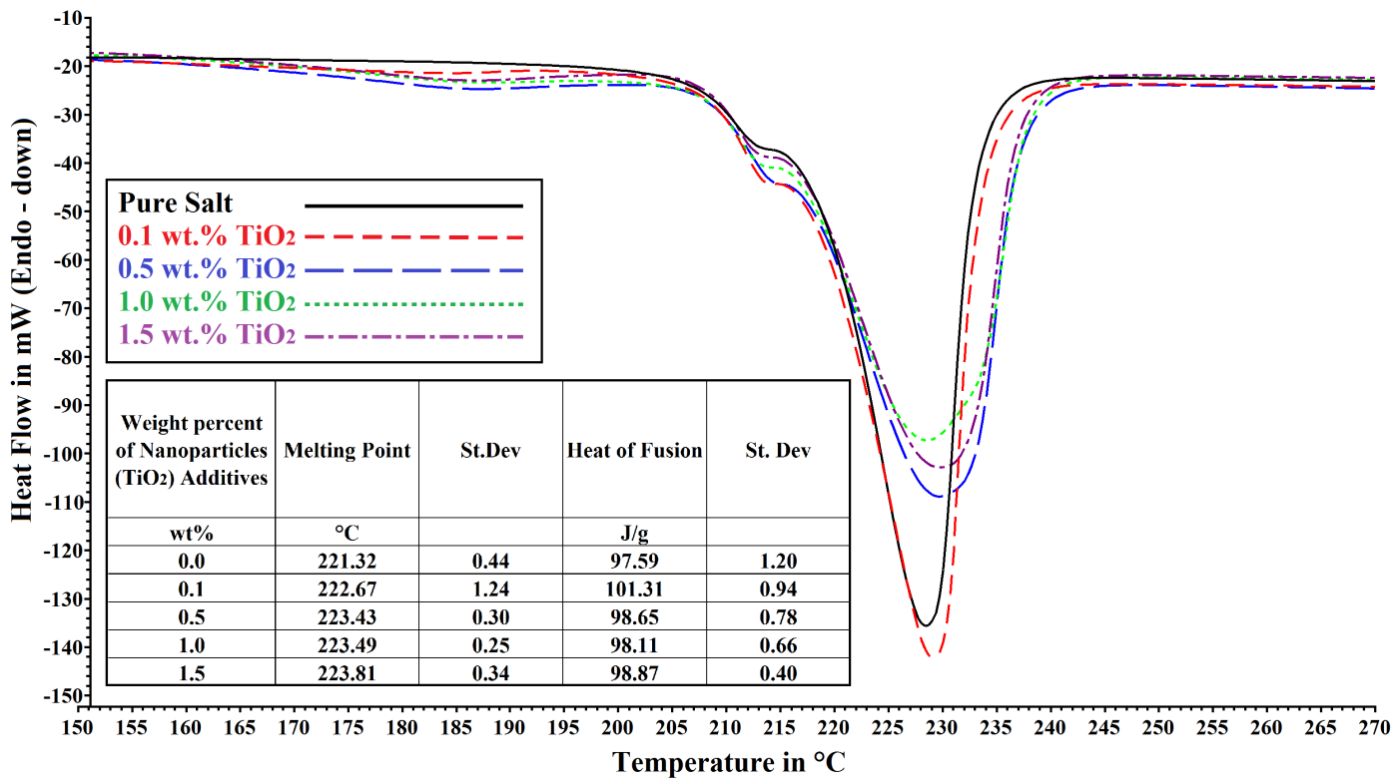
DSC curves of NaNO_3_ and KNO_3_ eutectic mixture (60/40) with and without TiO_2_ nanoparticles. Table inserted in the graph shows the average melting point and heat of fusion values.

The trend in the average heat of fusion of copper oxides decreases from 99.99 J/g with 0.1 wt % to 89.84 J/g with 1.5 wt %. The heat of fusion of 0.5, 1.0 and 1.5 wt % of TiO_2_ is very similar at ≈98.00 J/g when 0.1 wt % displays 101.31 J/g. It is not surprising to see such trend showing up as the dispersion of nanoparticles and the presence/size of agglomerate will affect both of these characteristics by altering the entropy of the medium. Whilst trend can be spotted, the relationship is still a difficult one to understand and it seems that large enhancement in the heat of fusion could forecast a high *C_p_* increase albeit the result from 0.1 wt % TiO_2_ goes against this statement. However the largest enhancements in enthalpy seen in Chieruzzi *et al*. were also the one where the rises in *C_p_* were the highest [32]. The formation of nano-structures whereby salt would be trapped inside tiny agglomerates could help understand this rise in enthalpy as more energy would be required to melt the solid trapped within these structures. The presence of nanoparticles (CuO and TiO_2_) within the molten salt would also lead to a broader melting range with a slight increase in the melting point. This is the case for TiO_2_ curves (small Table in Figure 3) but CuO does not fit this trend. Indeed for copper oxide, the melting point decrease from 223.86 °C for 0.1 wt % of CuO to 221.26 °C for 0.5 wt % and 220.66 °C when 1.0 wt % is utilized and then the melting point is back up to 224.12 °C at 1.5 wt % (small Table in Figure 2). This could be explained by a change in the amount and size of aggregate present as well as the way heat is transferred via nanoparticles (Diffuse or Ballistic) as the concentration of copper oxide rises from 0.1 wt % to 1.5 wt %.

**Figure 4:**
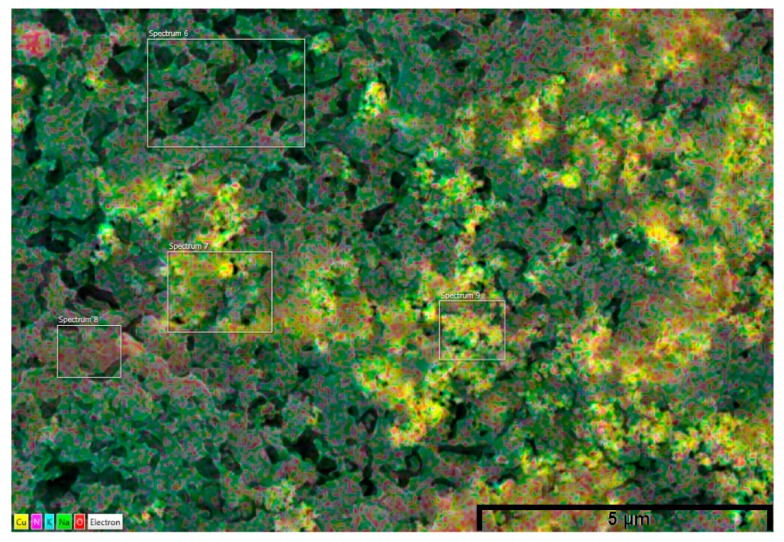
Energy dispersive X-ray (EDX) mapping of 0.1 wt % CuO (Yellow—Copper, Violet—Nitrogen, Blue—Potassium, Green—Sodium, and Red—Oxygen).

**Figure 5:**
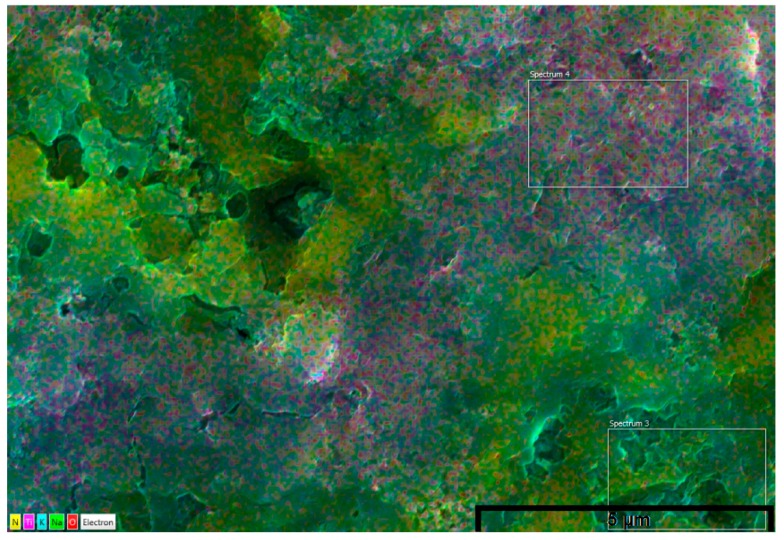
EDX mapping of 0.1 wt % TiO_2_ (Yellow—Nitrogen, Violet—Titanium, Blue—Potassium, Green—Sodium, and Red—Oxygen).

**Figure 6:**
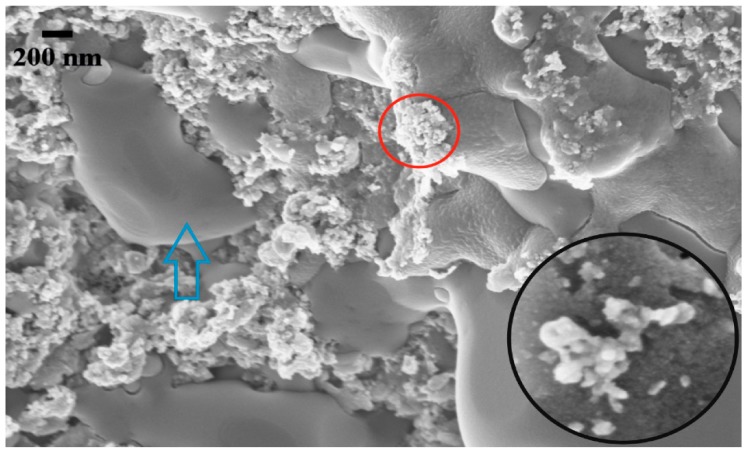
Scanning electron microscopy (SEM) image of 1.0 wt % Copper Oxide (×75,000 magnification). The blue arrow points at the surface of molten salt free from nanoparticles whilst the red circle shows agglomerate of CuO. Bottom right side shows a magnified image of copper oxide nanoparticles.

**Figure 7:**
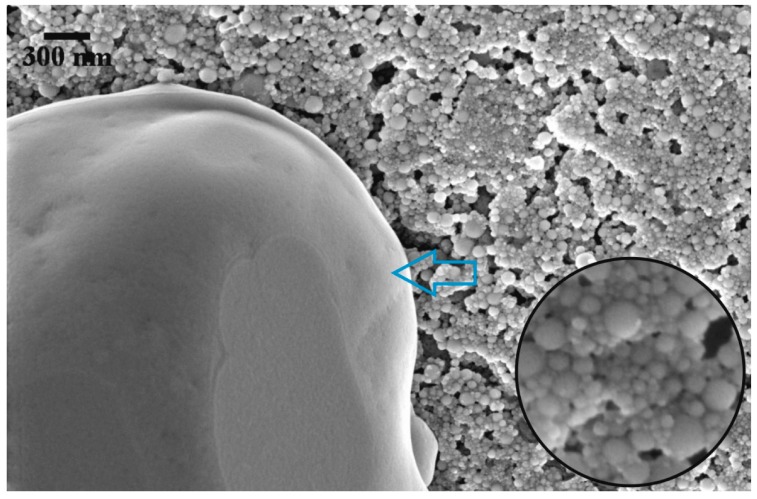
SEM image of 1.0 wt % TiO_2_ (×75,000 magnification). The blue arrow points at the surface of molten salt free from nanoparticles. Bottom right side shows a magnified image of titanium dioxide nanoparticles.

The dispersion of the nanoparticles was heterogeneous with large agglomerates seen both on the EDX mapping and on the SEM images. Various scanned spectrums showed that some areas where devoid of nanoparticles (Spectrums 6 and 8 on Figure 4, Spectrum 3 on Figure 5) whilst other showed large structure which looked to be interconnected with each other (Figure 4 and Figure 5—EDX Mapping—Yellow and Violet). Similarly the SEM images clearly show that some areas of the molten salt are free from nanoparticles (Blue arrow on Figure 6 and Figure 7) with other portions filled with big interconnected nanoparticles structures (Red circle on Figure 6). Lu and Huang, 2013 made the same observation with agglomeration of nanoparticles been easily distinguishable when Al_2_O_3_ was present ^25^. This was also showed and confirmed by Shin 2011 for SiO_2_ [28].

In this study both EDX and SEM clearly show agglomerated nanoparticles, which are similar to other publications [25,29,30,33,34]. Whilst it is possible to have well dispersed nanoparticles in liquid phase, the most likely scenario is the production of agglomerates within molten salt. The latter depending on their size will affect the energy density. Furthermore the shape of the nanoparticles is likely to affect the volume of nano-layers surrounding the particles. The TiO_2_ particles were spherical in shape with heterogeneous size distribution (Figure 7), whilst the CuO seemed to display a heterogeneous shape distribution whilst their size appeared to be more evenly distributed (Figure 6). The latter (Figure 6) displayed clusters of agglomerate unlike the TiO_2_ which seemed to show an interconnected layer of nanoparticles as shown on Figure 7. Could the production of small agglomerates be helpful toward the enhancement of *C_p_*? If that is so, then this variable would be a key factor in the definition of *C_p_*.

Furthermore, other variables such as loading factor, shape, size, density, homogeneity, surface charge and type will affect its behaviour in molten salt. With less than 20 published articles investigating this phenomenon, not enough data is available to try and devise a good model as to why this anomalous rise is observed.

In its liquid phase it is hypothesised that the enhancement in specific heat capacity comes from the interaction between the ionic liquid and the solid nanoparticles through the production of nano-layers. The latter would occur at the interface between the ionic liquid and the solid nanoparticle’s surface where the former behave as a semi-solid due to constraints surrounding the atoms. Because the enhancement in specific heat capacity cannot come from the nanoparticles themselves as the latter display lower values than the salt [35], the increase in *C_p_* could be explained by partially incorporating the enthalpy of fusion in this loosely parked semi-solid structure [31]. The production of structured layers at an interface with a solid has been observed Oh *et al.* research team where their research showed that liquid aluminium atoms at the interface with sapphire where constrained into layers [36].

A further two other modes might affect this anomalous enhancement in *C_p_*. First, the higher *C_p_* of nanoparticles themselves (compared to bulk) which has been reported by Tan *et al.* whereby the small particle size leads to the increased exposure of surface atoms [35]. The latter are less constrained and possess an excess volume in comparison to the atoms within its centre which as demonstrated by Wang *et al.* led to a blue shift in wave number for Al_2_O_3_ nanoparticles compared to bulk confirming this hypothesis [37]. Finally the interfacial thermal resistance could also have an impact on the enhancement of specific heat capacity due to an increase surface area to volume ratio provided by the nanoparticles if the latter are well dispersed. Whilst these three modes might each play a role in the enhancement of *C_p_*, it is believed that the formation of nano-layers would be the main driving force toward the anomalous rise in *C_p_* observed.

## 4. Conclusions

In this study, we investigated the dispersion of two types of nanoparticles in molten salt mixture (60 wt % NaNO_3_ and 40 wt % KNO_3_). The results showed that the variation in *C_p_* was dependent on the concentration for CuO (Table 1). However TiO_2_ behaved in a different manner showing no dependence on concentration with 0.5, 1.0 and 1.5 wt % concentration displaying similar enhancement in *C_p_* (Table 1). Furthermore the SEM data showed the production of clusters of nano-particle when CuO was present. This was not the case for TiO_2_ which showed a large interconnected layer rather than cluster. The shape and size distribution of the nanoparticles were very different which would impact its behaviour in the liquid phase. It is thought that the production of small agglomerates/nanostructures could have a beneficial impact on the enhancement of *C_p_*.

The development of heat-transfer-fluid in concentrated solar power plant application is an important field of research, as any enhancement in specific heat capacity would directly impact the storage capacity and price of production. As molten salt are one of the best alternative to synthetic oil, understanding how nanoparticles behave in such liquid might lead to the development of a more thermodynamically efficient HTF.
